# Organ-on-a-Chip and Lab-on-a-Chip Technologies in Cardiac Tissue Engineering

**DOI:** 10.3390/biomimetics11010018

**Published:** 2025-12-30

**Authors:** Daniele Marazzi, Federica Trovalusci, Paolo Di Nardo, Felicia Carotenuto

**Affiliations:** 1 Department of Clinical Sciences and Translational Medicine, University of Rome “Tor Vergata”, 00133 Rome, Italy; daniele.marazzi@uniroma2.it; 2 Department of Enterprise Engineering “Mario Lucertini”, University of Rome “Tor Vergata”, 00133 Rome, Italy; 3 Interdepartmental Center for Regenerative Medicine (CIMER), University of Rome “Tor Vergata”, 00133 Rome, Italy

**Keywords:** Organ-on-a-Chip, Lab-on-a-Chip, heart on a chip, elastomers, additive manufacturing

## Abstract

Microfluidic technologies have ushered in a new era in cardiac tissue engineering, providing more predictive *in vitro* models compared to two-dimensional culture studies. This review examines Organ-on-a-Chip (OoC) and Lab-on-a-Chip (LoC) platforms, with a specific focus on cardiovascular applications. OoCs, and particularly Heart-on-a-Chip systems, have advanced biomimicry to a higher level by recreating complex 3D cardiac microenvironments *in vitro* and dynamic fluid flow. These platforms employ induced pluripotent stem cell-derived cardiomyocytes (iPSC-CMs), engineered extracellular matrices, and dynamic mechanical and electrical stimulation to reproduce the structural and functional features of myocardial tissue. LoCs have introduced miniaturization and integration of analytical functions into compact devices, enabling high-throughput screening, advanced diagnostics, and efficient pharmacological testing. They enable the investigation of pathophysiological mechanisms, the assessment of cardiotoxicity, and the development of precision medicine approaches. Furthermore, progress in multi-organ systems expands the potential of microfluidic technologies to simulate heart–liver, heart–kidney, and heart–tumor interactions, providing more comprehensive predictive models. However, challenges remain, including the immaturity of iPSC-derived cells, the lack of standardization, and scalability issues. In general, microfluidic platforms represent strategic tools for advancing cardiovascular research in translation and accelerating therapeutic innovation within precision medicine.

## 1. Introduction

Biomedical research has traditionally relied on two-dimensional (2D) *in vitro* models and animal models to study human physiology and pathology. However, these approaches have inherent limitations that compromise their accuracy and clinical transferability. 2D cell cultures, typically grown on flat plastic surfaces, fail to faithfully replicate the complex physiological constitution of human tissues [1]. They lack the three-dimensional complexity of tissue, the influence of fluid flow, the intricate cell–cell interactions, and the crucial role of the extracellular matrix (ECM) that characterize the *in vivo* environment [2]. This fundamental limitation often leads to simplistic and ineffective models for studying drug response and disease mechanisms.

At the same time, animal models, although capable of recapitulating physiological processes *in vivo*, suffer from significant disadvantages. These include high costs, ethical aspects and, critically, poor transferability of results to humans due to inherent interspecies physiological differences [3]. For example, rat hearts (average body weight of 100−200 g) have a significantly lower heart mass (100−200 mg) than human hearts (250−300 g) and a significantly higher heart rate (400−600 beats per minute, BPM, at rest) when compared to human hearts (60−70 BPM at rest). This inherent physiological discordance, particularly at the electrophysiological level, limits their adequacy as a preclinical model for the evaluation of antiarrhythmic drugs. This translational gap often results in high failure rates and significant costs in clinical trials, making the drug development process expensive and inefficient [4,5].

To overcome these challenges, microfluidics emerged as an enabling technology. Microfluidics, the science of manipulating minute volumes of fluids (microliters to picolitres) within micrometer-scale channels (less than 1 mm wide), has become critical to addressing the limitations of traditional models [6]. This technology integrates principles of physics, chemistry, biology, and engineering, allowing precise control over fluid behavior, including laminar flow, diffusion-based mixing, and capillarity [7]. The shift to microfluidics represents a fundamental rethinking of *in vitro* experimentation, moving from large-scale static systems to dynamic micrometer-scale environments. This transition is not a simple miniaturization; It is a matter of achieving a previously unattainable level of environmental control [8,9]. Operating at the cellular and subcellular levels, microfluidics enables the precise recreation of conditions like those *in vivo*, such as shear stress and concentration gradients, which are crucial for cellular function and tissue organization [10,11]. This approach directly addresses the need for a deeper and less superficial discussion, allowing for more complex and relevant biological interactions.

This review examines the development and influence of advanced microfluidic technologies in *in vitro* biomedical research, with a particular emphasis on the OoC and LoC systems. Within the extensive range of applications, the focus is specifically on their role in cardiac tissue engineering and cardiovascular research, where the demand for human-relevant predictive models is especially critical. In the context of rapid advances in experimental platforms, the objective is to provide a critical and comparative evaluation of OoCs and LoCs, highlighting their design principles, shared technological foundations, and respective applications in the cardiac field. LoCs have enhanced analytical efficiency and high-throughput screening, laying the groundwork for more complex solutions; OoCs, by advancing the limits of biomimicry, aim to replicate the physiological functions of human tissues and organs with high fidelity. This comparison, with an emphasis on Heart-on-a-Chip models, elucidates the points of convergence and divergence between the two approaches, underscoring their synergistic role in overcoming the limitations of traditional preclinical models and advancing drug discovery, disease modeling, and personalized medicine in cardiology.

## 2. Organ-on-a-Chip (OoC)

### 2.1. Evolution and Biomimetic Principles in Organ-on-a-Chip

OoC technology emerged directly from the limitations of 2D cell cultures and animal models, which could not accurately mimic human physiology *in vivo*. A pivotal advancement occurred in 2011 with the study by Huh et al., which formalized the transition to biomimetic microfluidic systems capable of recapitulating tissue-tissue interfaces, chemical gradients, and mechanically active microenvironments [12].

OoCs are micro-engineered biomimetic systems that integrate cell biology, engineering, and materials science to reflect the structural and functional characteristics of human tissue. They place emphasis on recapitulating microarchitecture *in vivo*, aiming to mimic the three-dimensional (3D) organization of tissues, including cell–cell and cell-ECM interactions [13]. Recent work, such as the one carried out by Wnorowski et al., highlights how OoCs represent more predictive models of human physiology and pathological response, overcoming the constraints of traditional approaches [14]. Furthermore, the success of OoCs will also depend on the ability to integrate aspects related to micro-engineering, biomaterials, and in increasingly elaborate three-dimensional contexts, returning organ-like models of great fidelity [15].

A critical aspect of OoCs is the ability to recreate the dynamic environment *in vivo*, which includes:

- Fluid Flow and Shear Stress: Continuous perfusion ensures even nutrient distribution, removal of waste products, and the application of physiological shear stress, which is critical for maintaining cell viability and function for prolonged periods. This is a significant advantage over static crops [16,17].
- Mechanical Deformation/Stimulation: Many OoC models incorporate the mechanical forces (e.g., tensile stretching, compression) that cells experience *in vivo*. This is vital for the development, maturation and response of tissues to stimuli [18].
- Electrical Stimulation: Particularly in excitable tissues such as the heart, electrical signals are integrated to induce synchronized beating and promote tissue maturation [19].
- Biochemical gradients: Microfluidics facilitates the generation of precise biochemical gradients, allowing researchers to study cellular responses to drugs and signaling molecules in unprecedented detail [20].

### 2.2. Essential Components and Strategies of Bioengineering to Fabricate Organ-on-Chip

The integration of human-relevant cellular sources is critical for OoCs. Induced pluripotent stem cells (iPSCs) are revolutionizing personalized medicine by providing an unlimited supply of patient-specific cells [21]. When differentiated into specific cell types (e.g., iPSC-CMs), they retain the individual’s genetic information, allowing the study of patient-specific disease mechanisms, drug responses, and toxicity [22]. However, differentiation of iPSC cells can be inefficient and generate immature phenotypes which could potentially form teratomas or generate arrhythmias as in the case of cardiac tissues [23]. Primary cells, directly isolated from human or animal tissues, more closely represent the state *in vivo* and offer biologically accurate data. They are valuable for vaccine production, drug discovery, and gene therapy, and reduce reliance on animal models [21,24]. However, even if primary cells provide a physiologically relevant model and faithfully represent the state *in vivo*, they have some inherent limitations: they are generally more difficult to maintain *in vitro* for long periods of time, and they require strict culture conditions to preserve their functional characteristics [14]. Furthermore, adult cardiomyocytes (CMs) exhibit a very low proliferation rate, which hinders their isolation and culture [25].

Although immortalized cell lines present a cost-effective option due to their user-friendly nature and consistent availability, they exhibit considerable limitations when employed in Organ-on-Chip (OoC) models. Primarily, these cell lines fail to replicate the precise functional attributes of the target organ. Additionally, they are prone to genotypic and phenotypic alterations during experimental procedures, which may undermine the reproducibility of the outcomes and their correlation with clinical data. Furthermore, these cell lines are biologically homogeneous, thereby lacking individual variability that is intrinsic to human tissues [21,26]. In addition, in the case of immortal lines derived from primary renal cells, genetic and epigenetic changes (chromosomal reorganization, CNV) and progressive loss of phenotypic features, including the expression of key markers, are observed with negative impacts on their reliability to model renal physiology [27]. In specific models, such as the hepatic ones reported in the article by Dalsbecker, P. et al., immortal lineages differ genetically and metabolically from primary cells and, although they retain some functions (e.g., albumin secretion), exhibit lower expression of viable metabolic enzymes (such as CYP450), reducing their usefulness as accurate physiological models [28]. In Heart-on-Chip platforms, the AC16 (human ventricular), HL-1 (mouse atrial cardiomyocyte) and H9C2 (embryonic rat heart ventricle) cell lines are among the most commonly used cardiomyocyte models. These cell lines preserve the characteristics of differentiated cardiomyocytes while retaining their proliferative capacity [29].

Material selection for OoC devices balances functionality, fabrication, readouts, and biocompatibility. Common materials include polydimethylsiloxane (PDMS), glass, and thermoplastics (e.g., polystyrene (PS), poly (methyl methacrylate) (PMMA), polycarbonate (PC)). For HoC, materials must prioritize high biocompatibility, optical clarity, thermal conductivity [29,30].

The design and biochemical composition of scaffolds emulating the cardiac extracellular matrix are critical determinants of cellular behavior in HoC systems. Beyond providing structural support, the ECM delivers biochemical, mechanical, and topographical cues that regulate cardiomyocyte adhesion, alignment, lineage commitment, and functional maturation. To ensure physiologically relevant cardiac models, scaffolds must reproduce key features of the native cardiac microenvironment, including its molecular composition, stiffness, and anisotropic architecture [27,28,29,30,31]. Principal ECM constituents, including fibrous proteins (e.g., collagens, elastin) and glycosaminoglycans, play key roles in mediating cell–cell and cell–matrix communication and in activating signaling pathways that orchestrate cardiac tissue organization and functional development [32,33].

For example, as reported in the work of Goldfracht et al., it has been shown that the engineering of cardiac tissues, which mimics the biochemical and mechanical properties of the native ECM, is crucial to achieve the maturation and functionality of cardiomyocytes derived from hiPSC cells, emphasizing the importance of an optimal interaction between the cells and the scaffold [34,35,36].

It is crucial to recognize that mechanical, electrical, and topographical stimuli are necessary to achieve comprehensive structural and functional tissue maturation in HoC platforms [37].

Strategies to promote tissue maturation are constantly evolving. Mechanical conditioning, which involves the application of mechanical forces (e.g., cyclic stretching, stiffness gradients) to cardiac tissues in HoC models, promotes maturation, improving structural and functional properties such as contractility and force generation. Cardiomyocytes are highly responsive to environmental stiffness, with contractility increasing with increased stiffness [18,38]. Chronic electrical stimulation of iPSC-CMs in 3D cultures or HoC systems significantly improves maturation, leading to improved myofibril organization, conduction velocity, electrophysiological properties, and calcium management. This can lead to adult-like phenotypes, including a positive force-frequency response [39,40]. As reported by Ruan et al., the combined application of mechanical conditioning and electrical stimulation on iPSC-derived cardiomyocytes promotes structural and functional tissue maturation, improving myofibril organization, force generation, and electrophysiological properties, bringing the phenotype of the cells closer to that of adult cardiac tissue [41]. Finally, topographic stimuli and anisotropy are critical. Biomaterials with specific topographic structures and stiffness gradients drive cell alignment and promote maturation. Replicating the anisotropic (direction-dependent) architecture of native ECM, particularly in cardiac muscle, is crucial for coordinated electrophysiological and contractile activity [34,42,43].

The success of OoCs is not limited to providing static biomimetic stimuli, but extends to the creation of a dynamic feedback loop in which mechanical and electrical stimuli actively drive cell maturation, much like *in vivo* development [44,45]. This implies that the development of OoCs is not just about replicating a structure, replicating the developmental processes and dynamic interactions that lead to mature tissue function [46]. This adds a significant layer of complexity to the “simple” notion of a chip.

### 2.3. Organ-on-a-Chip Models and Applications in the Cardiac Field

OoC technology has led to the development of numerous organ-specific models, each with unique applications for disease modeling and drug screening.

OoC technology represents one of the most significant innovations in the field of experimental biomedicine, allowing the *in vitro* reconstruction of tissue microenvironments that reproduce with high fidelity the physiology and pathology of human organs [47,48]. Among the different models developed, Heart-on-a-Chip (HoC) stands out for its high potential in the study of cardiovascular diseases and in the preclinical evaluation of new therapeutic strategies. This device integrates micro-technologies and cardiac cell cultures in a dynamic system that allows the analysis of electrical, mechanical and biochemical parameters of myocardial tissue under controlled conditions [49,50]. An HoC typically consists of a microfluidic chip that allows continuous perfusion of the culture medium and the creation of chemical-physical gradients, micro-actuators capable of providing calibrated electrical or mechanical stimuli, and integrated sensors for real-time monitoring of tissue functions [51]. The biological compartment can include cardiomyocytes derived from induced pluripotent stem cells (iPSC-CMs), endothelial cells and fibroblasts, arranged in two-dimensional or three-dimensional configurations to reproduce the architectural and functional complexity of the human heart [52,53].

HoC offers a wide range of applications in the modeling of cardiac pathologies. Myocardial ischemia and infarction can be simulated by adjusting oxygen concentration or by using specific buffers to induce hypoxia, allowing for detailed analysis of electrophysiological and contractile changes associated with ischemic injury [54,55]. Cardiac fibrosis is reproduced by treatment with TGF-β or prolonged mechanical stimulation, allowing for investigating the remodeling processes and evaluating the efficacy of potential anti-fibrotic agents [56]. Localized scar lesions can be simulated through mechanical compression or laser micro-ablation, studying the mechanisms of functional compensation and the recovery of the surrounding tissue. Myocardial hypertrophy, induced by Angiotensin II or cyclic stretching, represents a further field of application, providing a reproducible model for the study of the cellular response to chronic overload stimuli [56,57].

The monitoring of the performance of a HoC is based on a series of quantitative readouts that allow an integrated assessment of the functional properties of cardiac tissue. Electrophysiological measurements, obtained by means of electrodes integrated in the chip, provide information on the contraction frequency, wavefront propagation and field potential variations, allowing the identification of arrhythmia or conduction abnormalities [29,58]. The contractility and strength developed by the tissue can be detected by high-resolution video microscopy or by means of integrated strain sensors [59]. For example, in advanced platforms, optical methods based on pixel correlation have been used to estimate the kinetics of the contraction of iPSC-CM at 250 Hz, or through “mini scope” platforms that record in real time the amplitude and duration of calcium transients in cardiac organoids [60]. The analysis of intracellular calcium transients, carried out with fluorescent probes, is a fundamental indicator of excitation-contraction coupling [61,62]. These parameters are accompanied by the quantification of specific biomarkers, such as troponin, BNP and pro-inflammatory cytokines, which allow us to correlate biochemical responses with functional ones [48,63].

In addition to disease modeling, HoC plays a central role in drug screening and precision medicine. The use of cardiomyocytes derived from individual patients’ iPSCs allows for capturing individualized responses to drugs and to predict, with greater accuracy, the risk of adverse events, as shown in patient-specific studies of trastuzumab and genetic variants that modulate susceptibility to anthracycline cardiotoxicity [64,65]. This is particularly relevant for the evaluation of the cardiotoxicity of chemotherapeutic agents such as doxorubicin and tyrosine kinase inhibitors, for which platforms with iPSC-CMs and/or HoC are allowed to define mechanisms of action and risk profiles with multi-omics and advanced imaging approaches [66]. Accumulated evidence suggests that these systems (especially when inserted into organ-on-a-chip or multi-organ workflows) can increase preclinical predictive capacity, bringing *in vitro* assays closer to real human heterogeneity. The high reproducibility and the possibility of integrating multiparametric analyses (electrophysiology, contractility, calcium transients, biomarkers) are further enhanced by the integration of sensors and actuators on the chip, elements that make the HoC a tool of choice for preclinical studies with high predictive content and compatible with screening [67,68,69]. Finally, multiple groups and industry reviews converge on the role of iPSC-CMs platforms in contemporary safety pharmacology and precision cardio-oncology, reaffirming the tool’s usefulness for risk stratification and guiding therapeutic decisions [70,71].

Although the Heart-on-Chip has unique characteristics, the development of models for other organs has also made relevant contributions to the cardiac context. Liver-on-chip models, for example, have shown how continuous perfusion and three-dimensional architecture can maintain specific functions for prolonged periods, an approach that can be transferred to long-term cardiac models [72]. Lungs-on-Chip have introduced cyclic mechanical stimulation methodologies that have inspired similar strategies to reproduce the physiological mechanical load of the myocardium. Kidney-on-Chip models, based on dual-channel systems, offer insights into the reconstruction of coronary perfusion and venous drainage, while Intestines-on-Chip, thanks to co-culture techniques with complex microbiomes, open perspectives for the study of interactions between heart and microbial metabolites [73,74].

The integration of HoC with other OoC systems gives rise to a multi-organ platform, often referred to as “body-on-a-chip” (BoC), which represents a frontier target with important implications for the study of systemic pathophysiological interactions and for the evaluation of complex pharmacokinetics. For example, multi-organ-on-a-chip models can simulate drug absorption, distribution, metabolism, and elimination (ADME) more realistically than conventional systems [75]. These systems use microfluidic solutions to connect modules representing different organs via simulated flow, allowing the reproduction of physiological crosstalk between tissues and signal modulation via arteriovenous reservoirs, which mimic the complete human blood circulation [67,76]. Such links allow *in vitro* measurement of PK/PD parameters such as Cm_ax_, area under the curve (AUC) and t_1/2_, opening new avenues towards the predictive and quantitative use of drugs as in the case of cisplatin [77]. The most recent studies confirm progress in the creation of multi-organ platforms oriented towards pre-clinical pharmacology, also in response to the needs that emerged during the pandemic. These models, although complex, are increasingly used to simulate systemic interactions and evaluate drug delivery dynamics in realistic scenarios [78]. In addition to the experimental aspect, the combination of multi-organ models with PBPK-PD models represents the next step to obtain *in vitro-in vivo* extrapolations, predicting more accurate responses in patients (IVIVE) [79].

Table 1 provides a comprehensive and immediate overview, summarizing the principal Organ-on-a-Chip models discussed within this section, along with their respective applications and the most utilized quantitative readouts. The comparative presentation of this information facilitates the rapid identification of structural and functional disparities among the various systems, underscoring the distinctive characteristics of the Heart-on-a-Chip relative to other micro-physiological platforms. This methodological framework supports a cross-sectional analysis of techniques and metrics, enabling the determination of strategies from other organ systems that may be transferable or adaptable to cardiac tissue studies. Nevertheless, the Heart-on-a-Chip encompasses numerous design and functional complexities, necessitating focused efforts for its optimization in both engineering and biological contexts.

### 2.4. Heart-on-a-Chip Engineering Optimization

The successful development of an efficacious Heart-on-a-Chip is contingent not only upon the selection of the cellular components and the optimization of stimulation parameters but also necessitates a precise engineering design of the entire system [97]. The selection of fabrication materials is a crucial element, as it affects not only biocompatibility, but also optical transparency, gas permeability, and mechanical properties of the chip [98]. Polydimethylsiloxane (PDMS) remains the most widely used material due to its elasticity and ease of micro-fabrication, but its tendency to absorb hydrophobic molecules has stimulated the adoption of alternatives such as thermoplastic polymers (such as PMMA, COC and PC) or glass-polymer hybrid materials, which provide greater chemical stability and reduced non-specific interaction with the drugs being tested [99,100].

Surface topography and substrate architecture play a decisive role in the functional maturation of cardiomyocytes. Micro and nano-structured patterns, obtained by photolithography or high-resolution 3D printing, orient cell alignment, favoring the formation of organized myofibrils and improving electrical coupling [101]. Studies have shown that micro-pattern surfaces with parallel lines or radial geometries increase electrical conduction velocity and contraction synchronization, bringing the behavior of the engineered tissue closer to that of the adult myocardium [102].

Stimulation strategies are often combined to promote model maturation and stability. Electrical stimulation, delivered through integrated micro-fabricated electrodes, induces more efficient cell–cell coupling and synchronous contraction, while cyclic mechanical stimulation, generated by flexible membranes driven by pneumatic chambers or piezoelectric actuators, reproduces the physiological load to which the heart is subjected *in vivo* [18,103]. In some cases, both forms of stimulation are applied simultaneously, achieving a synergistic effect on cardiomyocyte maturation and long-term tissue stability [104].

The most advanced platforms integrate multimodal sensors that allow simultaneous monitoring of electrical signals, contraction forces, and biochemical parameters [105,106,107]. Piezo-resistive and optical sensors are used to detect contraction-induced micro-deformations of the substrate, while electrochemical sensors allow in-line quantification of metabolites and markers of cell damage [108,109,110]. The adoption of high-resolution imaging techniques, including real-time fluorescence microscopy and optogenetics, also makes it possible to correlate subcellular events, such as calcium management, with macroscopic tissue function [68,111,112].

A further frontier is represented using three-dimensional bio-fabrication approaches to recreate portions of myocardial tissue in complex architectures, including integrated microfluidic vascular networks [113]. These configurations, obtained by bioprinting or casting in micro-fabricated molds, make it possible to guarantee a homogeneous supply of nutrients and oxygen, preventing areas of necrosis and allowing studies on cardiac models of greater thickness than traditional cellular monolayers. The integration of these solutions, together with optimized culture protocols and controlled stimulations, is progressively bringing the Heart-on-a-Chip closer to an *in vitro* model capable of faithfully reproducing not only the physiology, but also the biomechanical and electrophysiological complexity of the human heart [114,115].

### 2.5. Multi-Organ-on-a-Chip (MOoC) and Body-on-a-Chip Vision

Single-organ models, despite their utility, exhibit constraints in accurately simulating systemic reactions and the complex interactions between different organs. Consequently, Multi-Organ-on-a-Chip (MOoC) platforms have gained attention in recent research [116,117]. These platforms are engineered to incorporate multiple organ units that are fluidically interconnected, facilitating a comprehensive assessment and prediction of the systemic impacts of various compounds [118,119]. The vision of these systems is to interconnect individual organ-on-a-chip modules (including the heart) to create a single platform capable of mimicking all major organs, contemplating inter-organ scaling, common media and interdependent functionality [118]. MOoCs represent a paradigm shift, moving from the study of isolated organs to the understanding of complex systemic physiology and pathology. These systems will also allow us to understand how drugs are metabolized and how metabolites could cause off-target toxicity [120].

MOoC systems can mimic the process of Absorption, Distribution, Metabolism and Elimination (ADME) of drugs [121]. Liver-heart models are specifically designed to predict off-target cardiotoxicity after hepatic metabolism and demonstrate how the liver can mitigate or activate the effects of drugs such as cyclophosphamide and terfenadine. The involvement of tumor and cardiac modules in a heart–tumor chip allowed researchers to simultaneously test antitumor efficacy and cardiotoxicity, offering molecular insights on off-target effects [116,122]. Experiments conducted on a heart-kidney system have shown functional communication between cardiac and renal tissues in flow, opening new perspectives to describe systemic effects [123]. The gut-liver microchip features sections for intestinal, liver, and breast cancer cells to study absorption, liver metabolism, and antitumor activity. A chip with three organs (small intestine, liver and lung) has been used for pharmacokinetic studies of anticancer drugs [21]. A four-organ chip (gut, liver, skin, kidney) has been developed to test the systemic toxicity of drug candidates with stable homeostasis [124,125].

An example related to the systemic effect of hepatic metabolism on cardiac risks can be reported: the cardiotoxicity of compounds such as Terfenadine or Cisapride emerges or disappears as a function of CYP3A activity (or its inhibition), and this behavior has been reproduced in liver-heart platforms and in multi-organ systems with electrophysiological readings in the cardiac module and quantitative pharmacokinetic predictions at the “body-on-chip” level [116,126]. Similarly, integration with tumor modules has made it possible to model cardiac oncology on a chip, showing how doxorubicin induces damage in cardiac tissue in the presence of a metabolically active tumor compartment [127,128]. The vascular/robotic coupling of multiple chips and the scaling frameworks based on physiology and PB/PK today allow closed circuits with realistic flows, serial sampling and quantitative estimates of systemic exposure, creating the conditions to study heart–kidney, heart–liver and other pathophysiological interactions in a controlled manner [129,130].

**Figure 1:**
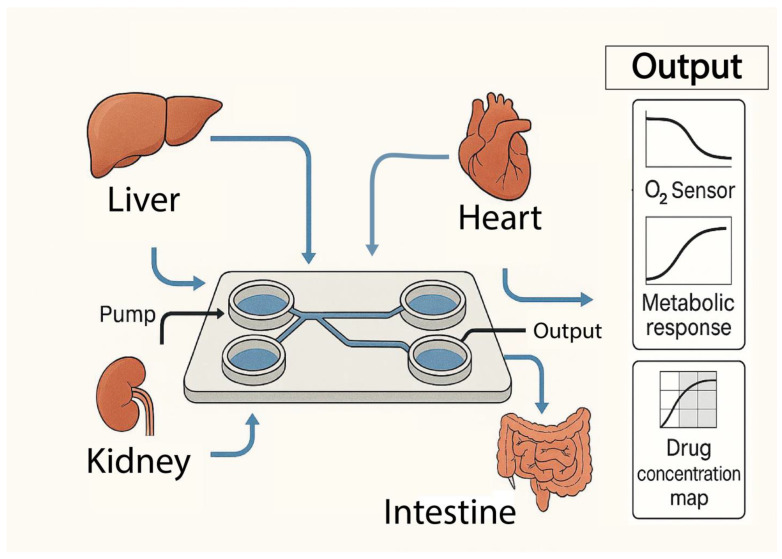
Schematic of a generic MOoC platform, showing interconnected organ modules via microfluidic channels for controlled perfusion and inter-organ communication.

Figure 1 shows a generic MOoC platform, highlighting the integration of multiple tissue-specific modules interconnected through microfluidic channels to enable controlled perfusion and inter-organ communication, and their outputs from integrated sensors monitoring biochemical, metabolic, and physiological responses.

## 3. Lab-on-a-Chip (LoC)

### 3.1. Fundamental Principles and Architectural Elements

**Figure 2:**
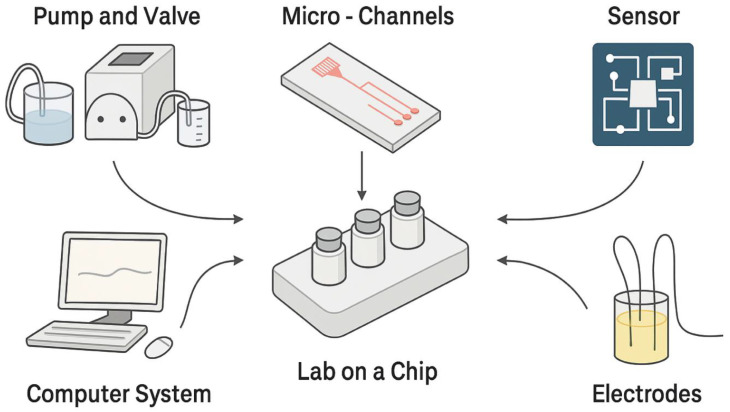
Overview of the components of a Lab-on-a-Chip, including microfluidic elements, control systems and experimental tools that allow the reconstruction of complex physiological conditions *in vitro*.

LoC devices are designed to condense and automate different laboratory functions on a single chip, offering distinctive benefits such as reduced fluid consumption, faster analysis times, superior process control, and increased compactness. These systems allow us to achieve high productivity, as they work in parallel, allowing the simultaneous execution of analyses and experimental phases, and consequently reducing production times [131,132]. Figure 2 shows the fundamental components that characterize a Lab-on-a-Chip system, including microfluidic units, regulation systems and experimental devices such as pumps, valves and electrical stimulators, designed to reproduce in a controlled and dynamic way the physiological conditions typical of tissues.

The core functionality of LoC devices is predicated upon fundamental principles of microfluidics. At this microscale, laminar flow predominates, characterized by the movement of fluids in smooth, parallel layers with negligible mixing across streamlines. This predictable flow behavior is crucial for the accurate manipulation of chemical reactions and transport processes [133]. As a result, diffusion-based mixing becomes the primary mechanism, as mixing occurs via molecular diffusion rather than turbulence, which can be a challenge for rapid mixing but also allows for the creation of stable concentration gradients [134]. The capillarity, where surface tension and wetting phenomena become dominant on a micrometric scale, allows the movement of the fluid without the need for mechanical pumps [135,136]. Electrophoresis, specifically electro-osmotic flow (EOF), is a mechanism that involves the application of an external electric field to induce fluid movement, acting on the free charges within the electric bilayer on the channel walls. EOF offers precise control over fluid movement and can generate a “plug flow” profile, which is almost planar, unlike the parabolic profile seen in pressure-driven flow [137,138]. Alternatively, pressure-driven flow involves applying gas pressure to a reservoir of fluid, pushing the liquid through micro-channels. This method offers fast response times, high stability, pulsation-free flow, and the ability to handle larger fluid volumes [139]. Modern controllers can integrate flow meters for precise flow control.

### 3.2. Manufacturing Methods

Photolithography is the fundamental basis for most LoC device fabrication processes, deriving directly from established techniques in semiconductor manufacturing [6,140]. Among the most common variants, soft lithography, which employs polydimethylsiloxane (PDMS), stands out for its simplicity of execution, high moldability, biocompatibility, gas permeability and optical transparency [141]. This technique allows the creation of fluidic microchannels using molds, making it particularly suitable for biological and biomedical applications [142]. 

In recent years, advances in 3D printing and laser engraving technologies have made it possible to overcome the limitations related to the complexity and high degree of specialization required by traditional micro-fabrication [143]. In this context, Stereolithography (SLA) emerges as a particularly relevant 3D printing technique for the fabrication of LoC and OoC devices, thanks to its ability to work with biocompatible printable elastomers. Elastomers are critically important materials for biomedical applications, as they can replicate the mechanical properties of biological tissues while offering high flexibility and biocompatibility [144,145]. 

The stiffness of these elastomers is quantified using the Shore A scale, which measures the hardness of flexible rubbers, from very soft to medium-soft, through a durometer that assesses indentation resistance [146]. Modulation of substrate stiffness is a critical factor for cell growth and function. Several studies have shown that the rigidity of the surrounding environment profoundly influences cellular behavior, including morphology, proliferation, differentiation, and contractility [147,148]. For example, cardiomyocytes are highly sensitive to extracellular matrix (ECM) stiffness, with contractility increasing as environmental stiffness increases [149]. Less mature cardiac cells, such as H9C2, show greater sensitivity to alterations in ECM stiffness than more developed neonatal cardiomyocytes, suggesting a crucial role of these stimuli in cell maturation [150,151]. The ability to fabricate elastomers with specific Shore A hardnesses (e.g., Shore A20 to Shore A60) allows for the creation of microenvironments that faithfully mimic the mechanical compliance of native tissues, directly influencing cell alignment, maturation, and contractility [152,153].

3D bioprinting offers considerable versatility in the processing of biomaterials with different physical properties and allows precise control over the three-dimensional organization of cell matrices [154]. The evolution from cleanroom lithography to affordable methodologies such as desktop 3D printing and laser engraving processes is democratizing the development of LoC devices. Using cost-effective LCD printers, it is now possible to fabricate microchannels and OoCs with high resolution at low cost [155,156]. This transition leads to a significant reduction in barriers to entry, which has historically been high due to equipment costs and the need for highly skilled personnel. As a result, a growing number of laboratories are now able to prototype autonomously and quickly, expanding the range of applications that can be explored and accelerating design and optimization cycles, with a direct impact on innovation in the sector [157].

### 3.3. Monitoring and Detection Methods

LoC systems integrate different sensing modes for real-time monitoring and data collection, which are essential for analytical accuracy and understanding of micro-scale biological phenomena.

Optical methodologies, encompassing fluorescence and chemiluminescence, are extensively employed due to their widespread instrumental accessibility and facile integration with microfluidic platforms. These techniques utilize the principles of light absorption, emission, or scattering to detect and quantify analytes, thereby providing numerous advantages, including high sensitivity, selectivity, and the capability to monitor biological processes in real-time [158,159]. Fluorescence, for example, allows extremely low detection limits, down to the single analyte, thanks to the high innate sensitivity of miniature optical sensors [160]. Chemiluminescence, on the other hand, offers very high sensitivity (up to biomarker picograms), with automated and rapid platforms such as the MDMF developed for troponin I [161]. Typical components include light sources such as LEDs and lasers, optical components such as micro-lenses and waveguides to optimize the light path, and detectors such as photomultiplier tubes (PMTs), CCD cameras, and CMOS sensors [162,163].

Electrochemical techniques, based on the measurement of current or voltage produced by Faradaic reactions, offer excellent sensitivity and are well suited to miniaturization and chip integration. Recent developments include organ-on-chip platforms that integrate electrochemical sensors for real-time monitoring of oxygen, glucose and lactate in 3D cultures [106]. Aptamer-based biosensors have been developed to detect cardiac biomarkers (such as CK-MB) secreted by heart-on-chip systems in response to cardiotoxic drugs [164]. Printed devices for electrochemical glucose detection demonstrated optimized sensitivity by enzymatic immobilization, with detection limits on the order of 0.01 mM [165]. Recent reviews highlight how electrochemical integration into LoC/OoC devices expands diagnostic customization and biopsy-liquid systems, as well as facilitating the monitoring of metabolites, biomarkers, and barrier parameters such as TEER [111,166].

Mass spectrometry (MS) is considered a preferred detection method for LoCs due to its high selectivity, sensitivity, and wide range of applications [167]. It can be interfaced with electrochemical reactors, often via pervaporation membranes, for continuous collection, identification, and quantification of volatile reaction products [168]. This configuration maximizes detection sensitivity and time response, allowing direct observation of the composition of the local reaction environment [169]. MS is particularly useful for the analysis of biomarkers and metabolites, providing a detailed profile of cellular and tissue responses in complex systems such as LoCs. They demonstrated the efficacy of a microfluidic platform coupled with mass spectrometry for rapid screening of enzymatic reactions, highlighting the potential of this technology in high-capacity biochemical research [170,171].

### 3.4. Wide Spectrum of Applications

LoC devices find applications in a wide range of fields, owing to their ability to miniaturize, integrate, and automate complex analytical processes within a single microfabricated platform. Their impact is particularly evident in clinical and biomedical sciences, where LoCs are increasingly employed for rapid pathogen detection, clinical diagnostics, forensic investigations, blood chemistry analysis, and protein and nucleic acid characterization [172,173]. In clinical practice, these systems enable point-of-care diagnostics by providing fast, cost-effective, and minimally invasive analyses, such as miniaturized microbiological cultures for digital dipsticks, as well as the diagnosis and monitoring of viral infections [174,175]. By minimizing assay duration and sample volume requirements, LoC technologies enhance accessibility and expedite medical decision-making, which is essential in emergency and resource-constrained environments. In the field of analytical chemistry, these technologies are transforming laboratory automation by consolidating multiple functions onto a single chip [176]. This degree of integration not only improves analytical throughput and reproducibility but also diminishes human error, reagent consumption, and operational expenses. Their adaptability to various assay formats renders them versatile platforms for a wide range of chemical and biochemical analyses [177]. Environmental sciences have also benefited from the advent of LoC devices, particularly through the development of total analysis systems (μTAS) for on-site detection of environmental hazards, toxins, and pollutants. Such portable and sensitive platforms provide timely monitoring tools for environmental safety and regulatory compliance, offering a significant advantage over conventional laboratory-based techniques, which are often time-consuming and logistically demanding [178]. Finally, in pharmaceutical research and high-throughput screening, LoC devices enable rapid and parallelized testing in synthetic chemistry and drug discovery workflows. By supporting miniaturized and automated screening of chemical libraries, they significantly accelerate the identification of lead compounds while reducing costs and improving experimental reproducibility [179].

### 3.5. HoC Integration in LoC Platform

LoC technology achieves its most transformative potential in cardiac tissue engineering through HoC platforms. These platforms adeptly integrate the microfabrication principles (Section 3.2) and sensing capabilities (Section 3.3) previously discussed in this chapter into cohesive systems capable of generating mature, functional myocardial constructs. Fundamentally, HoC devices utilize PDMS-based soft lithography or stereolithography-printed by GelMA hydrogels, which are calibrated to replicate the anisotropic stiffness and topography of native myocardium [180,181]. This approach facilitates the creation of scaffolds that actively guide the phenotypic maturation of iPSC-derived cardiomyocytes. These engineered substrates advance immature, fetal-like phenotypes, characterized by low contractile force, prolonged action potentials, and disorganized sarcomeres, toward functional, tissue-mimetic architectures that exhibit synchronized excitation-contraction coupling and adult-like electromechanical properties [182,183].

This structural priming sets the stage for dynamic physiological conditioning, where microfluidic perfusion establishes nutrient/waste gradients and supports paracrine signaling niches (e.g., via endothelial–cardiomyocyte crosstalk) in engineered tissues, while integrated actuators deliver the heart’s hemodynamic repertoire: cyclic uniaxial or biaxial strain to simulate preload and afterload, coupled with electrical field stimulation for chronotropic pacing [44,184]. Such combined microfluidic, mechanical, and electrical cues have been shown to enhance structural and functional maturation in heart-on-chip platforms, yielding contractile phenotypes closer to native myocardium [35,57,110]. Real-time interrogation via Section 3.3 modalities closes the loop: electrochemical impedimetric and apta-sensors quantify barrier function alongside biomarker kinetics (troponin I, CK-MB); multi-electrode arrays resolve action potential dynamics, restitution curves, and arrhythmogenic vulnerability; optical and CMOS platforms dissect contractility metrics, feeding data-driven refinements to scaffold composition, topography, and stimulation regimens [164,185].

HoC platforms directly address several fundamental constraints in cardiac tissue engineering. The incorporation of perfusable, endothelialized vascular networks overcomes diffusion limits at length scales exceeding 200 μm, thereby enabling angiocrine signaling and efficient metabolic coupling. Concurrently, the use of patient-specific, hiPSC-CM populations facilitates the detection of genotype–phenotype disparities, supporting precision pharmacological profiling in the context of cardiac channelopathies, hypertrophic and dilated cardiomyopathies, as well as drug-induced cardiotoxicity [13]. Nevertheless, substantial translational bottlenecks remain, including (i) in-complete metabolic reprogramming, particularly the transition from glycolytic metabolism to oxidative phosphorylation; (ii) dysregulated extracellular matrix remodeling characterized by an imbalance between matrix metalloproteinases and their endogenous tissue inhibitors; (iii) insufficient integration of immune and vascular signaling pathways; and (iv) limited scalability and parallelization of manufacturing processes to good manufacturing practice standards [98,186]. Addressing these challenges necessitates an explicit interdisciplinary convergence that integrates advanced mechanobiological modeling with the functionalization of bioactive scaffolds (e.g., enabling enzyme-mediated matrix remodeling), while strategically repositioning HoC platforms as the central nexus in which the miniaturization capabilities of LoC technologies are harnessed to drive clinically translatable myocardial regeneration [46,179].

## 4. Comparison Between LoC and OoC

### 4.1. Fundamental Divergence in Primary Objectives and Complexity

The fundamental distinction between LoC and OoC lies in their primary objectives and the inherent complexity of their design and application. LoCs are primarily versatile platforms for general analytical tasks [180]. Their primary goal is the miniaturization and automation of a wide range of laboratory functions for analytical accuracy [181]. This includes rapid pathogen detection, chemical analysis and high-throughput screening. Although they often involve biological samples, the focus is on efficient analysis rather than complex physiological mimesis [182,183].

In contrast, OoCs are specialized and biologically complex systems for physiological mimesis. The central mission of OoCs is to faithfully recapitulate human physiology *in vitro*. This requires the integration of living human cells into 3D organizations, the mimesis of tissue geometry, and their exposure to dynamic microenvironments [48]. The complexity extends to the incorporation of multiple cell types, ECM components, and physiological stimuli to obtain functional tissue units. The difference in the level of biomimicry and cellular complexity required is obvious: LoCs can operate with simpler cell types or even non-biological samples for analytical purposes [44,184]. OoCs, however, require human-relevant cells (often iPSCs or primary cells) and sophisticated bioengineering to achieve a high degree of biomimicry, including structural, mechanical, and functional aspects [185].

The key distinction lies in their design philosophy: LoCs aim for efficiency and scalability in analytical processes, while OoCs focus on fidelity and physiological relevance in biological modeling [13,186]. This difference brings about their respective complexities and challenges. Understanding this divergence clarifies why OoCs face unique biological obstacles, such as cell maturation, that are less central to LoCs’ targets [46,187].

### 4.2. Shared Technological Bases and Interdependencies

Despite their different purposes, LoC and OoC share common technological bases and show significant interdependence. Both are fundamentally based on microfluidics for the precise control of fluids, the management of small volumes and the creation of microenvironments with controlled and defined geometries [48]. Common fabrication techniques, such as photolithography, soft lithography (PDMS), and advanced 3D bioprinting, which allows the development of multilayer systems with different stiffnesses, are used by both technologies to create their intricate fluidic microstructures that simulate the in vivo environment [188,189]. Both platforms integrate various sensors for real-time monitoring and data collection. Optical (fluorescence, absorbance) and electrochemical detection methods are prevalent in both LoCs for general analysis and OoCs for monitoring cellular behavior, electrophysiology, and biochemical changes [190,191].

Advancements in LoC technology directly facilitate and propel the development of OoCs. In this context, OoCs can be regarded as a specialised application of LoC principles, which represents a significant advancement in the field of microfluidic engineering for biological purposes [30]. Innovations in microfluidic design, flow control, and sensor integration, developed within the broader LoC field, directly contribute to the growing sophistication and functionality of OoC systems [192]. For example, the ability to control shear stress and create stable gradients (fundamental principles of LoCs) is essential to mimic *in vivo* conditions in OoCs [107,193].

LoC and OoC should not be considered as separate entities, but rather as closely linked and interconnected systems, representing different declinations of microfluidic applications. LoCs provide the basic platforms, tools and methodologies for the manipulation and analysis of cellular systems at the microscopic scale, while OoCs push these technologies towards more complex applications, replicating specific functions and physiological conditions. In this way, a symbiotic relationship is established: methodological advances in LoCs fuel the evolution of OoCs, and the biological challenges faced by OoCs often stimulate technical innovation in LoCs, creating a virtuous circle of mutual advancement [194,195,196].

### 4.3. Complementary Roles in the Advancement of Biomedical Research

The choice between LoC and OoC, or their combination, depends on the specific objectives of the research. For high-throughput screening of many compounds with less emphasis on complex physiological interactions, a more generalized LoC platform may be sufficient [13]. Recent models suggest the applicability of microfluidics also to complex systems, such as miniaturized organismic models (e.g., *C. elegans*). As highlighted in the study by Yoon et al., the integration of these hybrid platforms enhances high-throughput screening and strengthens its correlation with in vivo physiology [197]. In contrast, for detailed mechanistic studies of disease progression, drug metabolism, or patient-specific responses, where in vivo relevance is critical, OoCs provide a superior platform [67,198]. In this context, an example of considerable relevance is the myocardium-on-a-chip model. As reported in the article by Ellis et al. (2017), the model allows cultivation of cardiomyocytes and endothelial cells, derived from a single strain of iPSC, in a 3D environment with capillary micro-vascularization for personalized and physiologically relevant studies [52]. The combination of both, for example using LoCs for initial high-throughput screening followed by OoCs for in-depth validation of promising candidates, offers a powerful integrated approach, optimizing both efficiency and physiological relevance [199,200]. An example of this integrated pipeline was provided by Phan et al., who developed a vascularized and perfused platform in a 96-well compatible format. Using this system to screen a library of compounds, they demonstrated how organo-mimetic devices can combine the physiological relevance of OoCs with the high-throughput workflows of LoCs [201].

However, the development of LoC devices and, even more so, OoCs remains complex and challenging. The implementation of such systems implies the need to overcome numerous obstacles, including: micro-fabrication (e.g., achieving high geometric precision, selection of appropriate materials, absorption of molecules by the material from which the device is made), fluid dynamics (e.g., obtaining uniform mixing and accurate flow control) and biological integration (e.g., promotion of cell maturation, stable multicellular co-culture, maintenance of long-term viability, and faithful replication of complex extracellular matrices) [202,203,204,205,206]. The lack of standardization across platforms further complicates widespread adoption and comparison [205]. These technologies are inherently interdisciplinary, requiring expertise in microfluidics, materials science, cell biology, tissue engineering, electronics, and computational modeling. The “simple” appearance of a chip hides the immense scientific and engineering rigor required to design, fabricate, and validate it for physiologically relevant results [203].

To advance the understanding of the fundamental attributes characteristic of microfluidic platforms, we present a comparative analysis in Table 2, which delineates the critical distinctions and convergences between the two microfluidic systems under examination. Table 2 offers a comprehensive elucidation of the respective advantages and limitations inherent to each technological paradigm. Furthermore, it identifies the most suitable applications, spanning from rapid high-throughput screening to complex mechanistic studies, and delineates the common features that facilitate the development of integrated methodologies. This synthesis provides an immediate and practical framework to guide experimental design processes.

### 4.4. Biological Limits and Translational Value of LoCs and OoCs

In addition to technological and engineering considerations, a crucial distinction between LoC and OoC platforms lies in their biological limitations, which directly influence their predictive power and translational value.

LoC models, while useful for analytical efficiency and high throughput, often use simplified biological systems, such as immortal cell lines or non-biological samples, to ensure reproducibility and scalability. However, this limits physiological relevance, especially in the cardiac context, where cellular heterogeneity, tissue dynamics, and electromechanical coupling are critical parameters [53,211,212]. Recent studies have shown that these limitations can reduce the predictivity of LoC models in cardiovascular pharmacology and in the study of inflammatory pathology [121].

OoC models integrate physiologically relevant human cell populations, such as iPSC-CMs and supporting stromal or endothelial cells, within three-dimensional, dynamically stimulated environments. These approaches allow them to reproduce more complex physiological phenomena than traditional 2D models, but present challenges related to the maintenance of long-term functionality and cell maturation [43,46,52]. iPSC-CMs often exhibit an embryonic/fetal phenotype, exhibiting electrophysiological and contractile properties that do not fully replicate those of the adult heart. In addition, maintaining viability and functionality in co-cultivation for extended periods is a significant challenge [205,206].

Inter-laboratory variability is a crucial challenge for the standardization of microfluidic models, resulting from differences in cell sources, differentiation protocols and the composition of the biomaterials or scaffolds used [100,213]. Such heterogeneity can significantly affect cell maturation, electrophysiological function, and contractile response of cultured cardiomyocytes, limiting the comparability of data across different laboratories [13]. As a result, while LoC models are often constrained by oversimplification of biology, OoC models face issues related to cellular immaturity, complexity of co-cultures, and difficulty in reproducing dynamic tissue interactions [214,215,216,217]. Both platforms thus highlight the fundamental challenge of replicating adult human cardiac physiology *in vitro* in a faithful and reproducible manner [218]. Addressing these biological constraints constitutes a fundamental prerequisite for the translational application of microfluidic models in cardiovascular research, as well as in the advancement of precision medicine strategies, which encompass predictive drug screening and individualized pathology investigations.

## 5. Conclusions and Future Developments

LoC technologies towards more complex systems such as OoC and MOoC represent a transformative advance *in vitro* biomedical research and personalized healthcare. These platforms, at the crossroads of engineering, biology and materials science, have moved beyond the role of simple microfluidic devices, establishing themselves as cutting-edge tools for the reproduction of human physiology in miniaturized and dynamic models.

The ability to faithfully mimic organ functions, incorporate patient-derived cells (e.g., iPSCs) and integrate with emerging technologies such as artificial intelligence is making OoCs powerful tools for understanding pathophysiological mechanisms, identifying new therapeutic targets, and optimizing treatment strategies from a precision medicine perspective. In addition, reducing reliance on animal models and improving predictivity rates in preclinical testing could contribute decisively to reducing costs and failures in the drug pipeline.

However, the effective transition to large-scale adoption requires overcoming challenges that are still open, including the standardization of protocols, the reproducibility of data, the integration of interconnected multi-organ modules on a chip, and the definition of clear and shared regulatory frameworks.

Future research on OoC technologies, particularly as applied to HoC systems, should primarily target the scientific and engineering constraints that currently limit their predictive accuracy and translational relevance. A central research priority is the development of HoC platforms that support statistically well-powered experimentation while maintaining high physiological fidelity. Realizing this objective will depend on advances in device architecture, stringent experimental standardization, and automated regulation of biochemical, mechanical, and electrical culture parameters, rather than solely on increasing experimental throughput.

Another critical line of investigation is the extension of HoC systems into MOoC configurations, in which the cardiac module serves as a central functional hub. In this context, research should emphasize the refinement of physiological scaling laws and dynamic inter-organ coupling strategies to more accurately reproduce systemic cardiovascular interactions. Careful consideration should be paid to elucidating how perfusion flow rates, compartmental volumes, and the temporal orchestration of stimuli modulate long-term cardiac function and affect the reproducibility and robustness of experimental outcomes.

From LoC standpoint, future efforts should focus on microfluidic architectures specifically engineered for cardiac applications. Key design features include precise spatiotemporal control of fluid flow, integrated electromechanical stimulation modalities, and on-chip sensing capabilities for continuous monitoring of electrophysiological, contractile, and metabolic readouts. The implementation of standardized, modular LoC platforms will be indispensable for enhancing reproducibility, facilitating protocol harmonization, and enabling meaningful cross-comparisons across independent HoC studies.

Finally, future HoC research should aim to improve tissue-level functional maturity and long-term stability by incorporating structured, multicellular cardiac microenvironments alongside advanced real-time monitoring and feedback-control strategies. In this framework, systematic integration of HoC platforms with computational modeling, machine learning, and other data-driven approaches constitutes a particularly promising avenue to interpret multiscale cardiac phenomena and to guide rational optimization of device design and operational parameters, thereby ultimately strengthening the predictive power of HoC-based preclinical models.

## Figures and Tables

**Table 1:** Representative Organ-on-a-Chip models: Main applications and readouts. The table summarizes the main OoC models discussed in the text, highlighting their specific applications and readouts used to evaluate tissue function.

OoC Model	Key Applications/Disease Models	Representative Quantitative Readouts
Heart-on-Chip	Ischemia–reperfusion, cardiac fibrosis, myocardial hypertrophy, LQTS, doxorubicin-induced cardiotoxicity, drug screening, personalized medicine	Electrophysiological signals (heart rate, rhythm, wavefront propagation, action potential amplitude/duration, resting membrane potential), contractile/kinetic beat force (contraction force, beat rate, calcium transient amplitude, post-rest potentiation), oxygen consumption, cell viability, cell morphology [80,81,82].
Liver-on-Chip	Drug metabolism, drug-induced liver injury (DILI), immune-mediated liver injury, chronic liver disease, ADME processes	Albumin secretion, expression of phase I/II enzymes and transporters (CYP activity), biomarkers of inflammation (interleukins, cytokines), fibrosis levels, transcriptomic analysis of specific cellular markers [83,84,85].
Lung-on-Chip	Pulmonary edema, impact of smoking, environmental particulate toxicity, air-blood barrier function, inflammatory responses	Production of intracellular reactive oxygen species (ROS), nanoparticle uptake, fluid accumulation (edema), endothelial cell activation, neutrophil adhesion/trans-migration [86,87,88].
Kidney-on-a-chip	Filtration, resorption, drug transport, nephrotoxicity, drug–drug interactions, regulation of homeostasis	Na/Pi co-transporter expression, albumin reabsorption, active transport of compounds (e.g., creatinine, PAH, metformin) via specific transporters (OCT2, OAT1), efflux ratios [26,89,90]
Gut-on-Chip	Nutrient/drug absorption, transport, gut-microbiome interactions, intestinal infections, inflammatory responses, peristaltic movements	Trans-epithelial Electrical Resistance (TEER) for barrier integrity, specific amino peptidase activity for epithelial function, excessive bacterial growth, secretion of inflammatory cytokines [91,92,93]
Brain-on-Chip	Modeling of the blood–brain barrier (BBB), neurodegenerative diseases (Alzheimer’s, Parkinson’s), drug transport through the BBB, neuroinflammation, neuronal communication	Trans-epithelial Electrical Resistance (TEER) for barrier function, permeability assays, electrical signals from neuronal networks (e.g., mean discharge frequency, synchronous discharge patterns), beta-amyloid aggregation, phosphorylated tau accumulation, neuro-inflammatory activity [94,95,96]

**Table 2:** Key Distinctions and Overlaps: Lab-on-a-Chip vs. Organ-on-a-Chip.

Characteristic	Lab-on-a-Chip (LoC)	Organ-on-a-Chip (OoC)
Primary Objective	Miniaturization and automation of analytical laboratory functions	Biomimicry of the physiology and pathology of human organs/tissues *in vitro* [13,48].
Biomimicry Level	Low-moderate; focus on analytical performance rather than complex *in vivo* replication	High; aims to replicate structural, functional and dynamic aspects of the native fabric [207,208]
Typical Cellular Components	It can use various cell types (primary, immortalized) or even non-biological samples; often 2D crops	Mainly human-relevant cells (iPSCs, primary cells); often 3D cultures with multiple cell types [7,209]
Key Application Focus	Diagnostics, analytical chemistry, environmental monitoring, general high-throughput screening	Disease modeling, drug discovery/toxicity/efficacy, personalized medicine, ADME-Tox, inter-organ communication [7,13]
Key Challenges	Manufacturing complexity, surface-dependent effects, signal-to-noise ratios, fluidic actuation	Cell maturation, long-term culture viability, standardization, material limitations (PDMS uptake), chronic disease reproduction, inter-organ scaling [13,208,209]
Typical outputs/readouts	Chemical concentrations, presence/absence of analytes, electrical signals, optical signals	Electrophysiological signals, contractile strength, biochemical markers (e.g., albumin, enzymes), barrier integrity (TEER), cell morphology, gene expression [7,48,210]

## Data Availability

No new data were created.
